# Source attribution of human echinococcosis: A systematic review and meta-analysis

**DOI:** 10.1371/journal.pntd.0008382

**Published:** 2020-06-22

**Authors:** Paul R. Torgerson, Lucy J. Robertson, Heidi L. Enemark, Junwei Foehr, Joke W. B. van der Giessen, Christian M. O. Kapel, Ivana Klun, Chiara Trevisan

**Affiliations:** 1 Section of Epidemiology, Vetsuisse Faculty, University of Zürich, Switzerland; 2 Department of Paraclinical Sciences, Faculty of Veterinary Medicine, Norwegian University of Life Sciences, Adamstuen Campus, Norway; 3 Department of Animal Health and Food Safety, Norwegian Veterinary Institute, Oslo, Norway; 4 Vetsuisse Faculty, University of Zürich, Switzerland; 5 Center for Zoonoses and Environmental Microbiology, National Institute of Public Health and the Environment, Netherlands; 6 Department of Plant and Environmental Sciences, Faculty of Science, Section for Organismal Biology, Denmark; 7 Centre of Excellence for Food- and Vector-borne Zoonoses, Institute for Medical Research, University of Belgrade, Belgrade, Serbia; 8 Department of Biomedical Science, Institute of Tropical Medicine, Belgium; International Atomic Energy Agency, AUSTRIA

## Abstract

**Background:**

A substantial proportion of echinococcosis transmission to humans via contamination of food has been assumed. However, the relative importance of food as a transmission vehicle has previously been estimated through expert opinion rather than empirical data.

**Objective:**

To find and evaluate empirical data that could be used to estimate the source attribution of echinococcosis, in particular the proportion that is transmitted through contaminated food.

**Methods:**

A systematic review was undertaken to identify reports on the risk factors for human cystic (CE) and alveolar (AE) echinococcosis. Data bases searched included PubMed, Scopus, Web of Knowledge, Cab Direct, Science Direct, Google Scholar, eLIBRARY.RU, CyberLeninka, CNKI and VIP. Search terms included Echinococc*, hydatid, epidemiology, logistic regression, risk factors, odds ratio, relative risk, risk factors. Reports, including grey literature where available, that had suitable data were selected and data were extracted. The main pathways of transmission were hypothesised to be contact with the definitive host, contaminated water, contaminated food and contaminated environment (other than food). For each study the attributable fraction for these potential sources of infection was calculated from the data presented. A meta-analysis was then undertaken to obtain pooled estimates for the relative contribution of these transmission pathways.

**Results:**

Data from 28 cross-sectional studies and 14 case-control studies were extracted. There was strong evidence for transmission by direct contact with dogs for both CE and AE. The estimated attributable fractions were 26.1% (CI 13.8%-39.6%) and 34.4% (CI 20.7% -48.2%) respectively. Transmission through contaminated water was estimated to be responsible for approximately 29.4% (CI 12.1%-51.7%) for CE and 24.8% (CI 10.6% to 42.6%) for AE. Contaminated food may be responsible for approximately 23.4% of CE cases (CI 2.1%-47.3%). Globally, there was insufficient evidence to conclude AE can be transmitted by food, although case control studies from low human incidence areas suggested that possibly 32.5% (CI 10.0%-53.2%) could be transmitted by food. There was also insufficient evidence that direct contact with foxes was a significant source of human disease. There were no suitable studies with a risk of environmental contact reported, but the residual attributable fraction thatwould likely include this pathway was approximately 21.1% for CE and 11.1% for AE.

**Conclusions:**

The results support the hypothesis that dog contact and drinking contaminated water are major pathways of transmission of both CE and AE. For contaminated food, the results are less consistent, but suggest that it is an important transmission pathway and provide better evidence than expert elicitations as previously used.

## Author summary

*Echinococcus granulosus* and *E*. *multilocularis* are zoonotic parasites that cause human cystic (CE) and alveolar (AE) echinococcosis, respectively: both diseases resulting in a substantial burden of disease. They are transmitted to humans via wild or domestic caniid definitive hosts. This study aimed at finding and evaluating the source attribution of echinococcosis and provides evidence that transmission by direct contact with the definitive hosts perhaps results in 26.1% and 34.4% cases of CE and AE, respectively. Indirect transmission by contaminated water may result in 29.4% and 24.8% of cases of CE and AE, respectively. There is evidence that indirect transmission through contaminated food may result in 23.4% of cases of CE globally. Contaminated food may result in 32.5% of cases of AE, but only in low incidence regions such as Europe. In areas of high human incidence such as China, the evidence for foodborne AE was not convincing. Other sources of transmission such as contact with a contaminated environment result in approximately 21.1% of CE cases and 11.1% of AE cases.

## Introduction

Human echinococcosis is a parasitic disease in which people are infected with the larval (metacestode) stage of tapeworms belonging to the genus *Echinococcus*, acting as aberrant, dead-end intermediate hosts. The two most common forms of human echinococcosis are cystic echinococcosis (CE), which is caused by the larval stage of several host-adapted species of *Echinococcus granulosus senso lato* and alveolar echinococcosis (AE), which is caused by *E*. *multilocularis*. The usual definitive host of *E*. *granulosus* is the domestic dog [1], whereas various species of fox (*Vulpes* spp.), are the usual definitive host of *E*. *multilocularis* [2]. However, dogs are also highly susceptible to infection with *E*. *multilocularis* [3] and, in some endemic regions, may be important in transmission. Both these parasitic diseases have been hypothesized to be transmitted, at least partially, to humans through contaminated food. A multi-criteria ranking of foodborne parasites in Europe suggested that *E*. *multilocularis* is the most important foodborne parasite in this region, with *E*. *granulosus* in 4^th^ place [4]. The global burden of disease attributed to contaminated food has been estimated to be 40,000 (95% uncertainty intervals 16,996–322,953) Disability Adjusted Life Years (DALYs) and 312,000(9,083–640,716) DALYs annually for CE and AE, respectively [5]. Although the total burdens of disease caused by echinococcosis were estimated or modelled on empirical evidence, the proportions of these diseases that were foodborne were estimated on the basis of an expert knowledge elicitation [6,7]. This elicitation suggested that the foodborne proportion of the global burden of these diseases was a median of 21% for CE and 48% for AE. However, the confidence intervals (CIs) for the estimates were wide and, in the case of AE, were so wide as to be largely uninformative, with between 1% and 76% (95% CI) of AE estimated to be foodborne. Nevertheless, it is often the median that is cited in reports, whereas the actual burden that is foodborne remains unknown. An opinion produced by the European Food Safety Authority [8] noted the difficulties in estimating the relative importance of foodborne pathways in the transmission of these parasites, but also stated that although there are difficulties in estimating the extent to which foodborne transmission occurs, the potential for food to act as a transmission vehicle is incontrovertible [8].

There is no doubt that untangling the various pathways of infection for humans by *Echinococcus* spp., and the relative importance of each, remains a challenge. Although direct contact with infected definitive hosts would seem an obvious route, there are reports of cases of human echinococcosis where there has been no contact with the definitive host or where contact with dogs is not a risk factor. For example, in a community in North West China, Wang et al. [9] found virtually the same prevalence of CE in dog owners and in people who did not own dogs. Echinococcosis is a chronic disease and several years may elapse between infection and the onset of clinical symptons [10] making epidemiological studies a challenge. Furthermore, even where these diseases are endemic, the actual numbers of human cases are often low. This means it can be difficult to estimate source attribution from such studies. However, in theory it should be possible to estimate attributable fractions (AF) due to various risk factors from data from cross-sectional studies, as the prevalence of disease, the proportion of individuals exposed to a particular risk factor, and the risk ratio (RR) and odds ratio (OR) can be estimated. Case-control studies can also be used by assuming that the OR is a reasonable estimate of the RR as the prevalence of disease is low.

In an attempt to provide better empirical evidence for the various sources of echinococcosis in humans, we have undertaken a systematic review to locate studies from which population AF can be calculated. As a second stage, a meta-analysis was undertaken to estimate the AF due to contact with dogs or other definitive hosts, ingestion of contaminated drinking water, consumption of contaminated food, or contact with the contaminated environment, these being the likely pathways of infection for humans. Participants were subjects involved in either cross-sectional studies or case-control studies investigating risk factors for echinococcosis. Outcomes from these studies were RR or OR for various risk factors for echinococcosis. From these results and other data reported it was possible to estimate the AF for these major pathways of transmission for each study and a pooled estimate across all studies.

## Materials and methods

### Systematic review

The systematic review was compiled based on the PRISMA guidelines [11] (supporting information S1 checklist).

Principal data sources selected to carry out the literature search included the bibliographic databases: PubMed, Scopus, Web of Knowledge, Cab Direct, Science Direct and Google Scholar, the Russian databases eLIBRARY.RU, CyberLeninka, and the Chinese databases CNKI, VIP. The bibliography of articles found in these searches were also assessed for additional reports. This included searches for grey literature. The computer search was not constrained by language or date, although the eligibility criteria were restricted to 5 languages: English, Spanish, standard Chinese, Russian, and Arabic. Search terms included Echinococc*, hydatid, epidemiology, logistic regression, risk factors, odds ratio, relative risk, risk factors. [Echinococc*, OR hydatid AND (epidemiology OR logistic regression OR risk factors OR odds ratio OR relative risk OR risk factors)]. The key words were also translated into the 5 languages and searched for in the appropriate databases. For example, searches in Spanish included Factores de Riesgo para Equinococ*, or in Russian Фактор Риска Эхинокок*.

### Selection criteria

Titles were examined to indicate whether each document might contain information on risk factors for infection with *Echinococcus* spp. The abstracts and full texts of selected articles were then examined further. Those that included statistical analysis of risk factors associated with AE or CE were selected for potential data extraction. Only articles that used imaging data or confirmed surgical cases as a basis for diagnosis of infection were selected for data extraction. Documents where only serology was used for diagnosis were excluded as serology is too unreliable to use in such population studies [12]. A small number of studies were also excluded as they originated from areas where both AE and CE are endemic, but there was no diagnostic differentiation between the two diseases; i.e. patients were classified as having echinococcosis rather than CE or AE. Data on OR, RR or quantitative data on subject numbers and exposure to various risk factors were extracted. Those reports that had interpretable data from which an AF could be estimated were deemed to be of sufficient quality to be included. There was no restriction with regard to time, so all years were considered.

### Data extraction

Data on OR and, where available, numbers of echinococcosis cases associated with each risk factor in the study were extracted. Data from which the OR were estimated were extracted and these were used to estimate the RR and the proportion of the population potentially exposed to the risk factor. For case-control studies, the OR was used as an estimate of the RR (see below).

### Estimation of attributable fraction from extracted data

AF can be estimated from the total numbers of cases within a population minus the number of cases for which that risk factor is not associated. The AF is, therefore, the proportion of disease attributed to that particular factor, or the proportion by which the disease would be reduced if that risk factor were to be removed. Some studies indicated negative AF and these can be interpreted as protective factors; i.e. if the risk factor were to be removed then the disease prevalence would increase by that proportion. In the absence of actual case numbers in the reports, these numbers can be estimated from the OR, the standard error of the log OR, and the proportion in the population that had echinococcosis. For case-control studies, the OR was used as an estimate of the RR, on the assumption that the disease prevalence was low.

Studies often had multiple risk factors and it is possible that the total AF could add up to more than 100%. This is because some risk factors are not mutually exclusive (e.g., gender and dog contact). Therefore, AF were estimated for 4 broad categories representing potential transmission pathways: (1) direct contact with an infected dog; (2) contamination of water supplies; (3) contamination of food; and (4) other, which is essentially environmental contamination leading to transmission. In addition, direct contact with foxes was investigated for AE. Although there are other definitive host for *E*. *multilocularis* such as the racoon dog [3], there were too few studies investigating these hosts as a risk factor for human AE to enable any meaningful analysis. These transmission pathways were assumed to be mutually exclusive and hence AF would add up to 100%. Other factors, such as gender or age, were not considered as these were not mutually exclusive. Thus, dog care could be associated with gender: one gender might be more likely to look after dogs (so it is the dog that is the transmission pathway). Also increasing age is often associated with an increased incidence of echinococcosis because of the greater time exposed via any transmission pathway.

Different studies also did not investigate risk factors in a standardized way. For example, risk factors associated with a potentially infected dog could be described as “dog ownership”, “contact with dogs”, or even “regular treatment of dogs” or “feeding dogs offal”. Thus, inclusion of data for the most appropriate risk factor associated with dogs from each study for further analysis had to be decided on a case-by-case basis.

In each study the OR or RR was used for the appropriate exposure pathway from the multivariable analysis. Otherwise it was reported from the univariable analysis or calculated from data presented.

### Meta-analysis

All analyses were undertaken in R [13].

For the data sets arising from a cross-sectional study design, the AF was estimated according to:

$$AF=\frac{p_{i}\{{RR}_{i}-1\}}{1+p_{i}\{{RR}_{i}-1\}}$$ (1)

Where RR_i_ is the RR of disease as estimated from the data extracted from the ith study, and p_i_ is the estimated prevalence, also from the data extracted, of the risk factor in the population of the ith study.

For case-control studies, the RR and prevalence of the risk factor in the population are unknown. However, because the population prevalence of echinococcosis is low, then it is possible to use the OR as an approximation of the RR. Similarly, the prevalence of the risk factor in the population is approximately equal to that of the prevalence of the risk factor in the control group. This is because even if the diseased group has a much higher prevalence of exposure to the risk factor, because of the rare disease assumption, the contribution to the population prevalence of the risk factor in diseased individuals will be negligible.

Thus, for case-control studies:

$$AF\approx \frac{p_{ic}\{OR_{i}-1\}}{1+p_{ic}\{OR_{i}-1\}}$$ (2)

where P_ic_ is the proportion of controls that are positive for exposure to the risk factor in the ith study and OR_i_ is the OR of disease in the ith case-control study for the risk factor of interest.

Using the package metaphor, the pooled RR (including the OR as an estimate for the RR in case-control studies) across the studies and its standard error were estimated. A random effects model was used to incorporate heterogeneity across studies into the analysis. Possible publication bias [14] was corrected for using the trimfill function. Likewise, it was possible to estimate the pooled logistic transformation of *p*, and this was again adjusted for possible publication bias. To estimate the pooled AF, bootstrapping was used. Here, 1000 random samples of the log of the pooled RR were taken, with the estimate of the log(RR) and its standard error, from the rnorm function in R. Similarly, 1000 samples of each of the logit of p were estimated. Both the log(RR) and logit(p) samples were back transformed to the linear scale and 1000 samples of the AF were then estimated from formula (1). From this calculation, the mean and 95 percentiles were extracted to estimate the pooled AF and 95% CI.

For each selected study, the AF was estimated from either Eq (1) or (2) and 95% CI were estimated by inputting the extracted data (2x2 table) and generating 1000 replicates using a Dirichlet random number generator (from the MCMCpack package) and thus generating 1000 replicates of the AF. The CI were estimated from the 2.5 and 97.5 percentiles of these replicates.

These CI from both the individual studies and from the bias corrected pooled estimates, together with the midpoint estimates, were used to generate forest plots.

For each study, a meta-analysis of the OR for the selected risk factors was undertaken and again corrected for possible publication bias.

## Results

**Fig 1:**
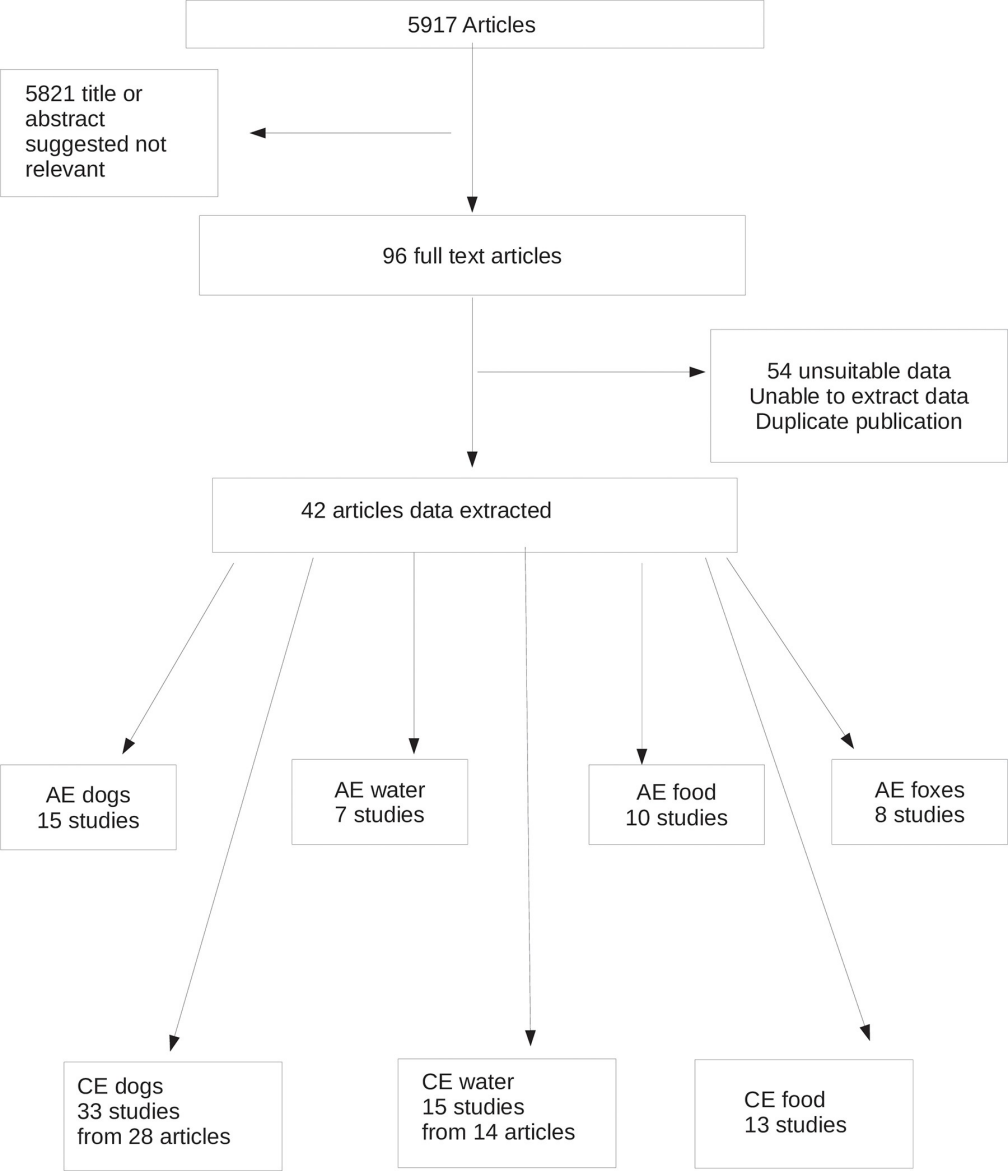
Prisma flow diagram with the search strategy steps.

**Fig 2:**
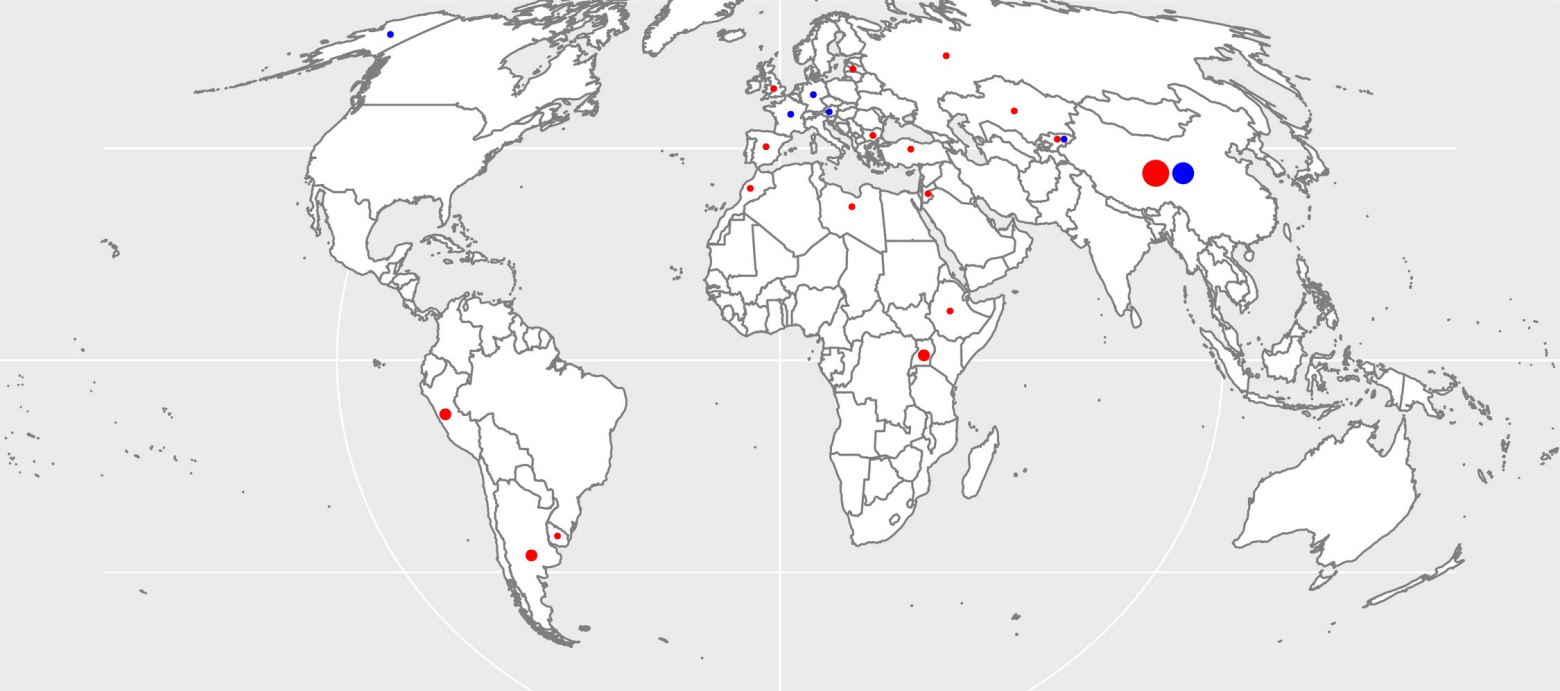
Geographical distribution of studies. For CE (red, size proportional to number of selected studies) this ranged from 17 studies from China, 2 from Argentina, Uganda, and Peru, and 1 for other countries. For AE (blue), there were 10 studies from China, and one each from Kyrgyzstan, Germany, Austria, France and USA (Alaska). The map was generated using the open source software R [13] using a shape file downloaded from Natural Earth (www.naturalearthdata.com).

The systematic search gave a total of 5917 articles (Fig 1). Following reading of the title and/or abstract, 96 full text articles were identified that appeared to have data of interest. Of these, 54 had unsuitable data, incorrect study design, or it was not possible to extract the data. This left 28 cross-sectional studies and 14 case-control studies from which it was possible to extract data (references [15–56]). Many of these had data for both diseases and/or several risk factors. Fig 2 illustrates the numbers of studies found and the geographical location where the studies were undertaken. For CE, there were 27 cross-sectional studies involving 85,181 individuals with 2129 being diagnosed with CE (2.5%). There were 9 case-control studies that examined in total 721 cases and 997 controls. Four of these studies had a control: case ratio of 1:1, three with a ratio of 2:1 and two with a ratio of 3:1.

For AE, there were 10 cross sectional studies with suitable data. These combined data represented 24,950 study subjects of which 1014 were found to have AE. This gave a prevalence of AE across these studies of 4%. For the case-control studies there were 295 cases and 847 controls across all 5 studies, two studies examined 3 controls for every case, two examined 4 controls for every case and one examined 5 controls for every case with a mean of 2.9 controls for every case.

### Estimated attributable fractions

**Fig 3:**
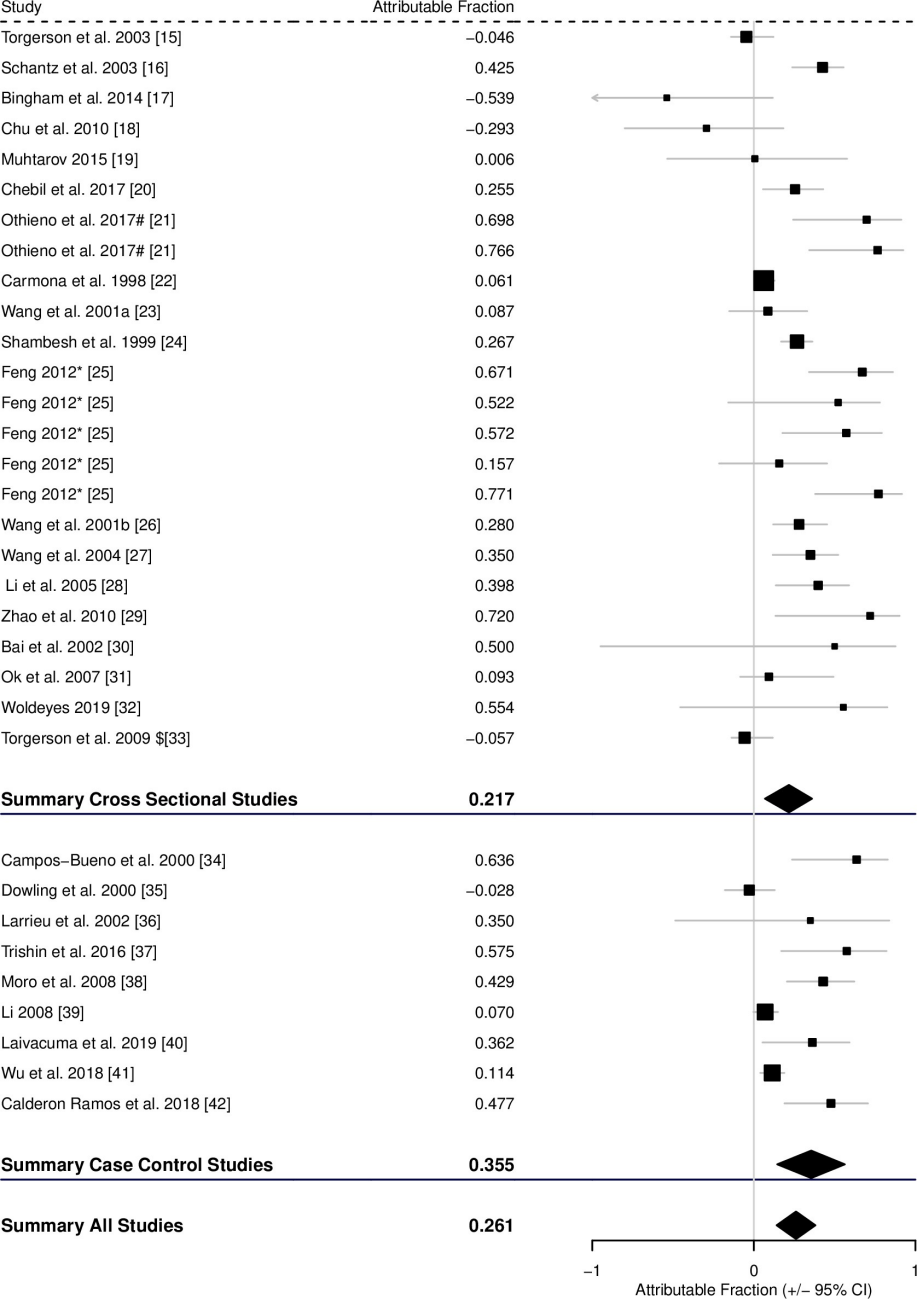
Forest plot for the attributable fraction for 24 cross-sectional studies and 9 case-control studies that analysed potential contact with infected dogs as a potential risk factor for CE. * and # indicate different studies from the same published report. $ Data not reported in study, but supplied by the authors.

For the AF of CE associated with possible contact with an infected dog, 24 cross-sectional studies had useful data to estimate the AF and a further 9 case-control studies. The pooled AF fraction, calculated from the corrected pooled RR and pooled OR, across the cross-sectional studies was 0.217 (0.069–0.360), across the case-control studies was 0.355 (0.145–0.563), and across all studies was 0.261 (0.138–0.396) (Fig 3, Table 1).

**Table 1:** Estimated AF as the source of infection for cystic and alveolar echinococcosis and corresponding OR following meta-analysis of selected studies.

	Cystic Echinococcosis		Alveolar Echinococcosis	
Factor	AF (CI)	OR (CI)	AF(CI)	OR(CI)
**Dog contact**	0.261 (0.138–0.396)	2.504 (1.765–3.557)	0.344 (0.207–0.482)	1.982 (1.592–2.469)
**Water contamination**	0.294 (0.121–0.517)	3.061 (1.700–5.514)	0.248 (0.106–0.424)	2.073 (1.468–2.974)
**Food contamination**	0.234 (0.021–0.473)	1.817 (1.047–3.151)	0.157 (-0.199–0.456)	1.315 (0.767–2.255)
Food contamination (no correction)*	0.071 (-0.113–0.289)	1.193 (0.709–2.009)		
**Fox contact**			0.140 (-0.065–0.384)	1.300 (0.872–1.935)
**Residual**	0.211 (-0.061–0.488)		0.111 (-0.301–0.522)	

*AF and OR when analysis are not corrected using the trim and fill method

**Fig 4:**
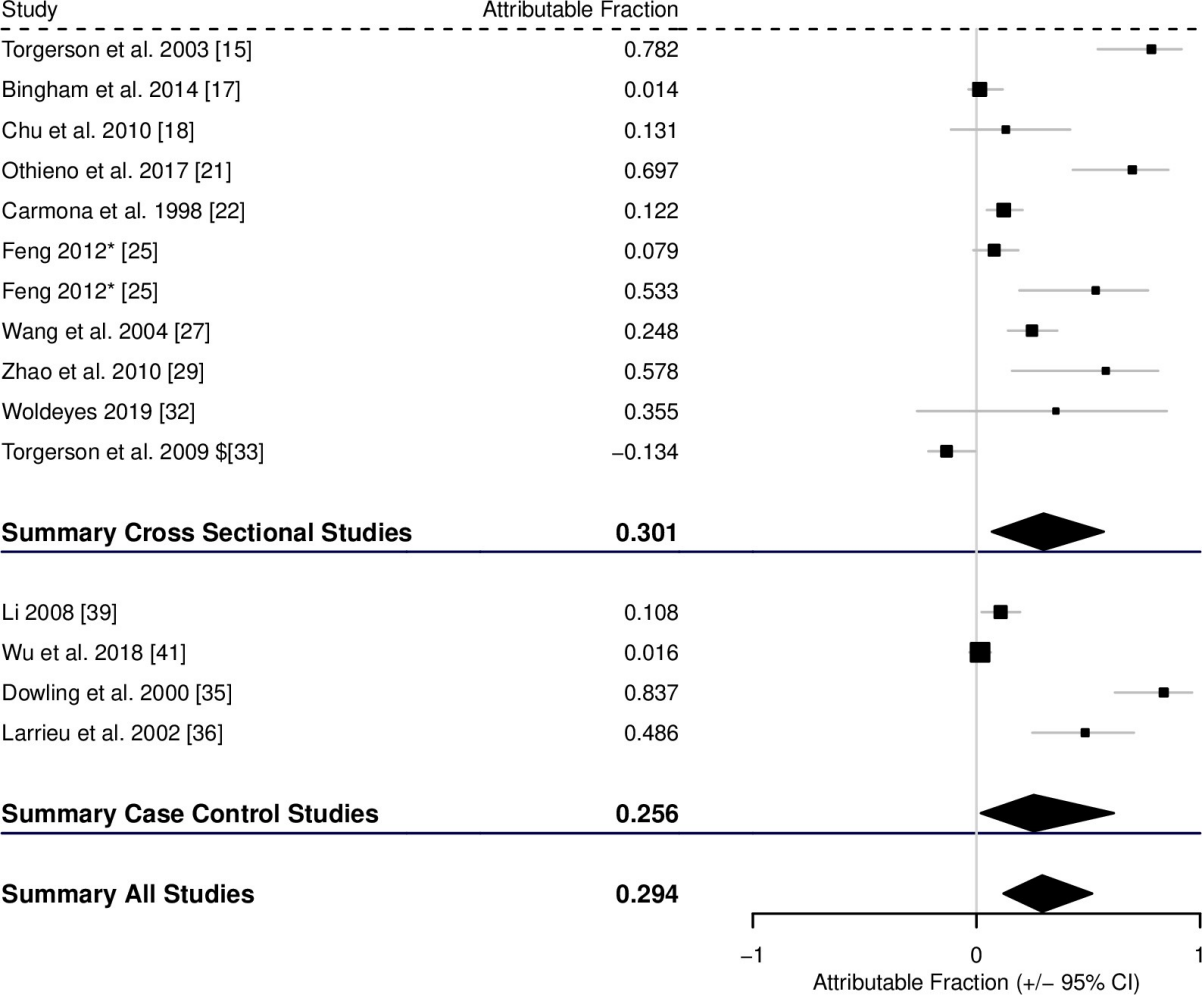
Forest plot for the attributable fraction for 11 cross-sectional studies and 4 case-control that analysed contaminated water as a potential risk factor for CE. $ Indicates different studies in the same published report the same source. *Data not reported in study, but supplied by the authors.

The AF associated with contaminated water data was extracted from 11 suitable cross-sectional studies and a further 4 case-control studies. Results suggest an estimated AF of 0.300 (0.069–0.569 from the cross-sectional studies, 0.256 (0.020–0.614) from the case-control studies, and across all studies 0.294 (0.121–0.517) (Fig 4, Table 1).

**Fig 5:**
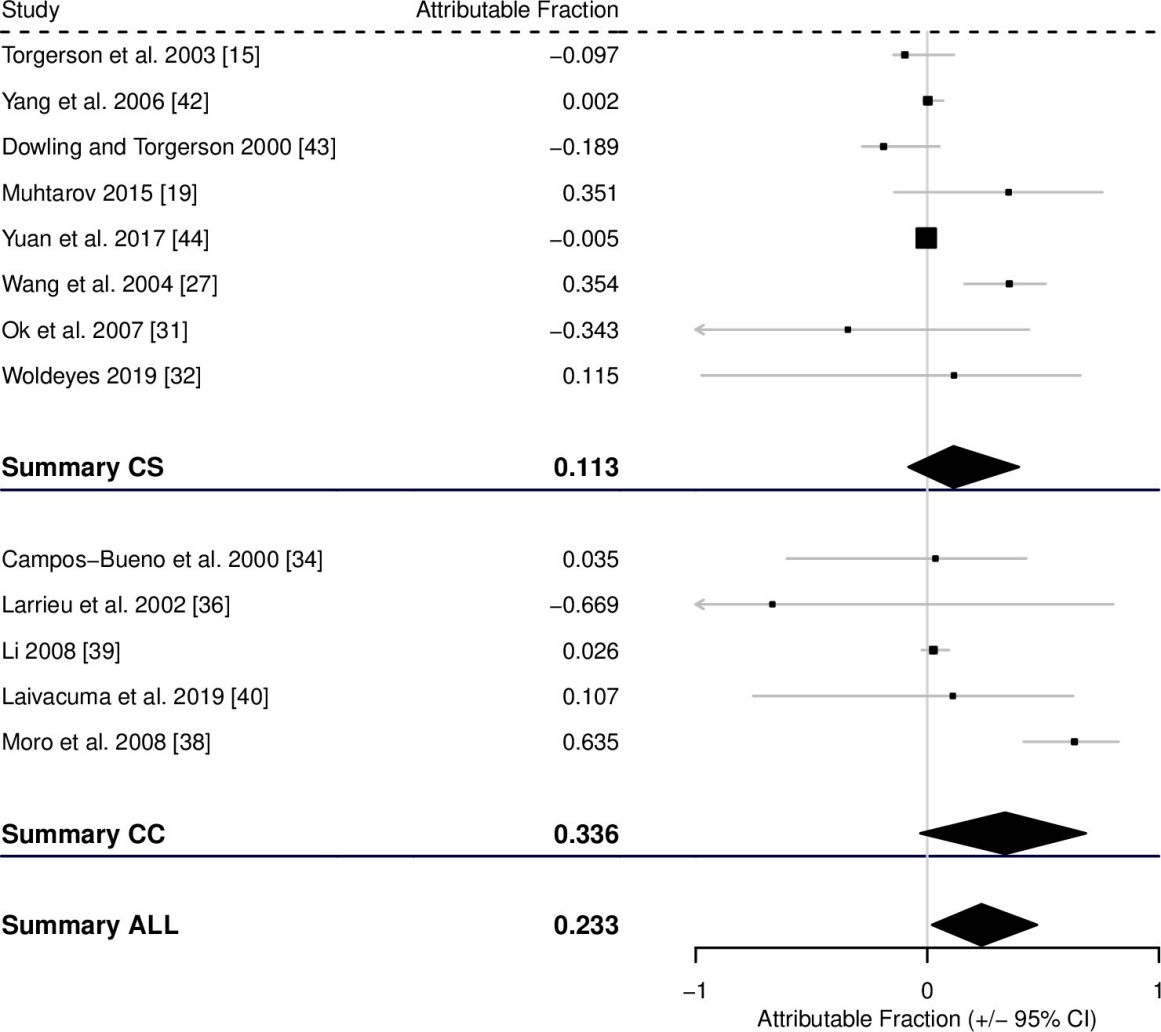
Forest plot for the attributable fraction for 8 cross-sectional studies and 5 case-control studies that analysed contaminated food as a potential risk factor for CE.

**Fig 6:**
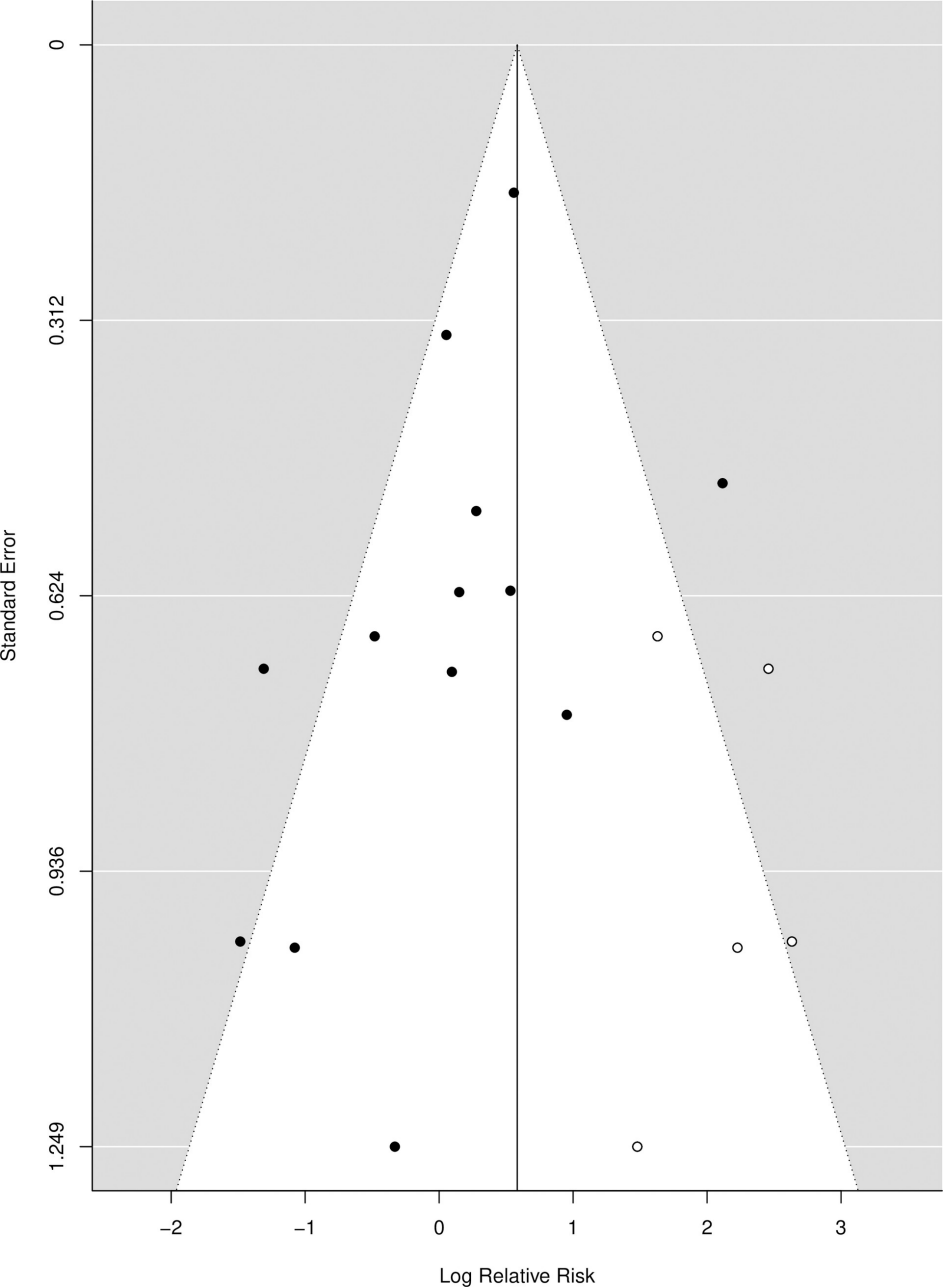
Funnel plot illustrating the relative risk (or OR as an estimate of RR) of studies of CE associated with food. Black circles are the selected studies, and open circles represent missing data.

The AF associated with contaminated food data was extracted from 8 suitable cross-sectional studies and 5 case-control studies. Results suggest an estimated AF of 0.113 (-0.081–0.395) from the cross-sectional studies, 0.336 (-0.030–0.684) from the case-control studies, and 0.234 (0.021–0.473) across all studies (Fig 5, Table 1). However, there was substantial evidence of bias that resulted in adjustments using the trim and fill method. Without such adjustments, the pooled AF across all studies was 0.071 (-0.113–0.289). The funnel plot (Fig 6) illustrates the data adjustment with the trim and fill method.

**Fig 7:**
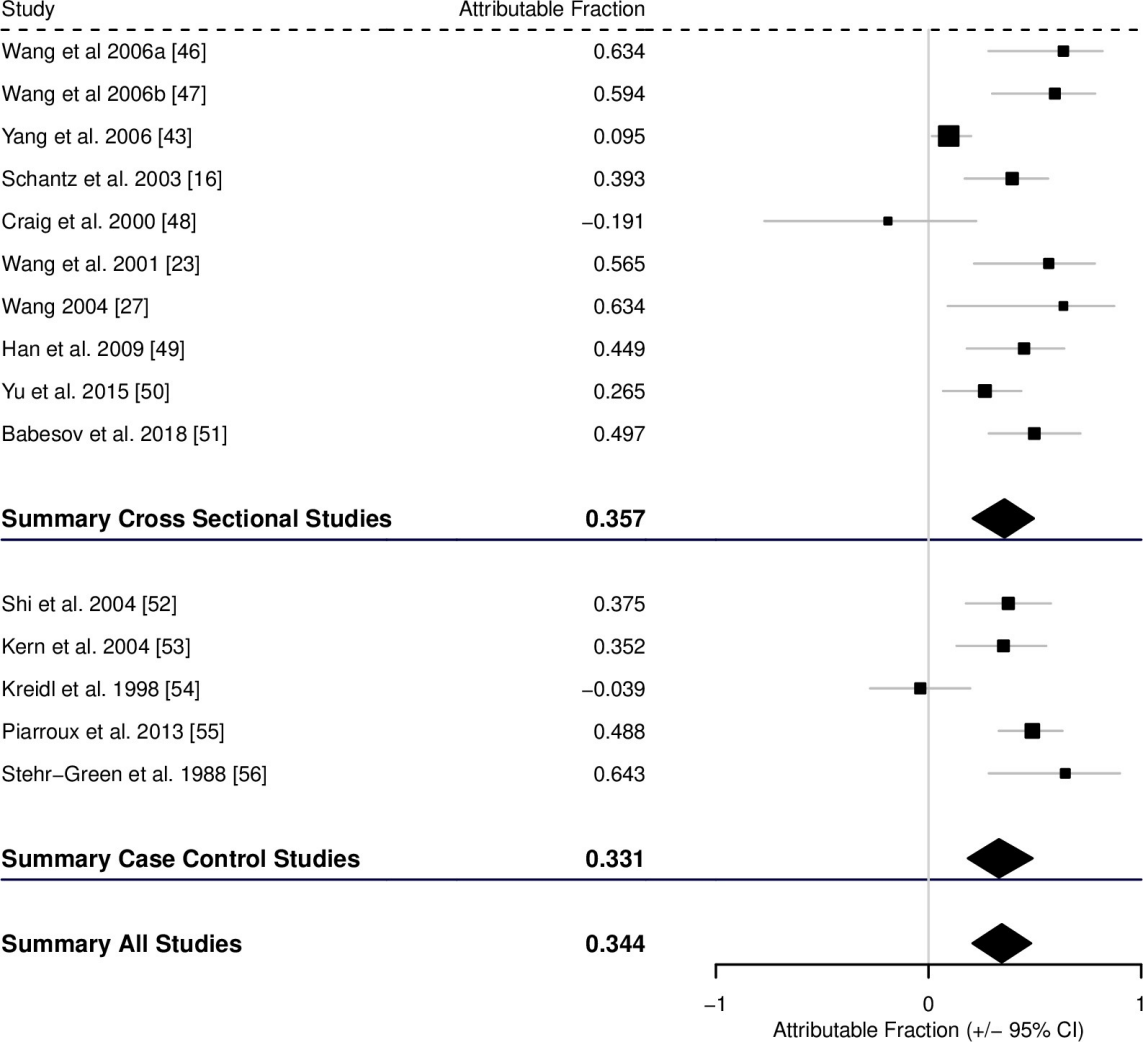
Forest plot for the attributable fraction for 10 cross-sectional studies and 5 case-control studies that analysed contact with infected dogs as a potential risk factor for human AE.

Fewer suitable studies were found for alveolar echinococcosis. For contact with potentially infected dogs there were 10 suitable cross-sectional studies and 5 case-control studies. The AF was estimated to be 0.357 (0.208–0.495) from the cross-sectional studies, 0.331 (0.185–0.489) from the case-control studies, and 0.344 (0.207–0.482) across all studies (Fig 7, Table 1).

**Fig 8:**
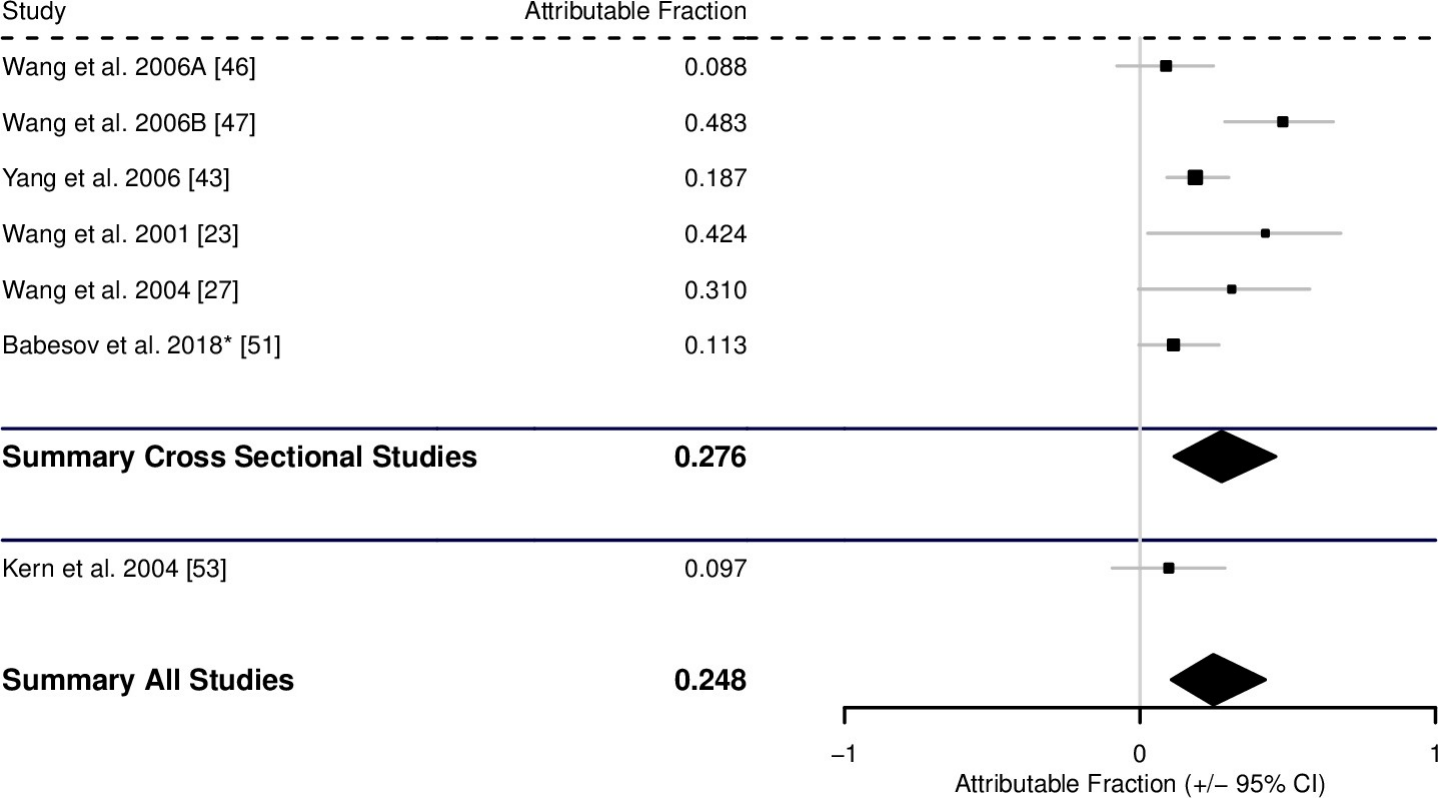
Forest plot for the attributable fraction for 6 cross-sectional studies and 1 case-control study that analysed contaminated water as a potential risk factor for human AE. *Data not reported in study, but supplied by the authors.

A total of 6 cross-sectional studies and 1 case-control study had suitable data for analysis of the AF associated with contaminated water for AE. These 6 cross-sectional studies suggest an AF of 0.276 (0.115–0.424), the single case-control study had an estimated AF of 0.097 (CI -0.094–0.287) giving a pooled AF across all studies of 0.248 (0.106–0.424) (Fig 8, Table 1).

**Fig 9:**
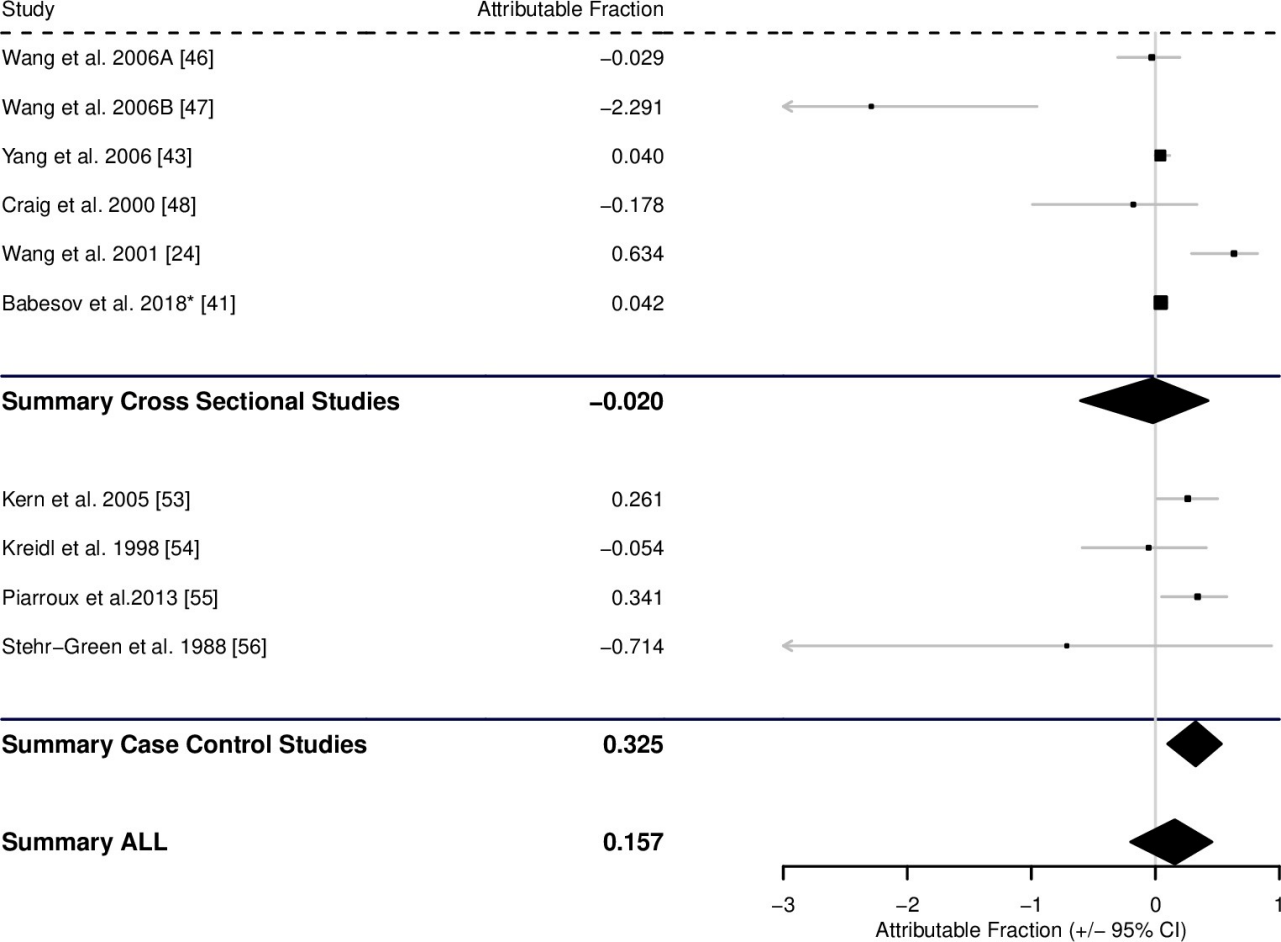
Forest plot for the attributable fraction for 6 cross-sectional studies and 4 case-control studies that analysed contaminated food as a potential risk factor for human AE. *Data not reported in study, but supplied by the authors.

The 6 cross-sectional studies that analysed contaminated food as a risk factor for AE suggested an AF of -0.020 (-0.604–0.425), whereas the 4 case-control studies suggested an AF of 0.325 (0.100–0.532), giving an overall pooled estimate of 0.157 (-0.199–0.456) (Fig 9, Table 1).

**Fig 10:**
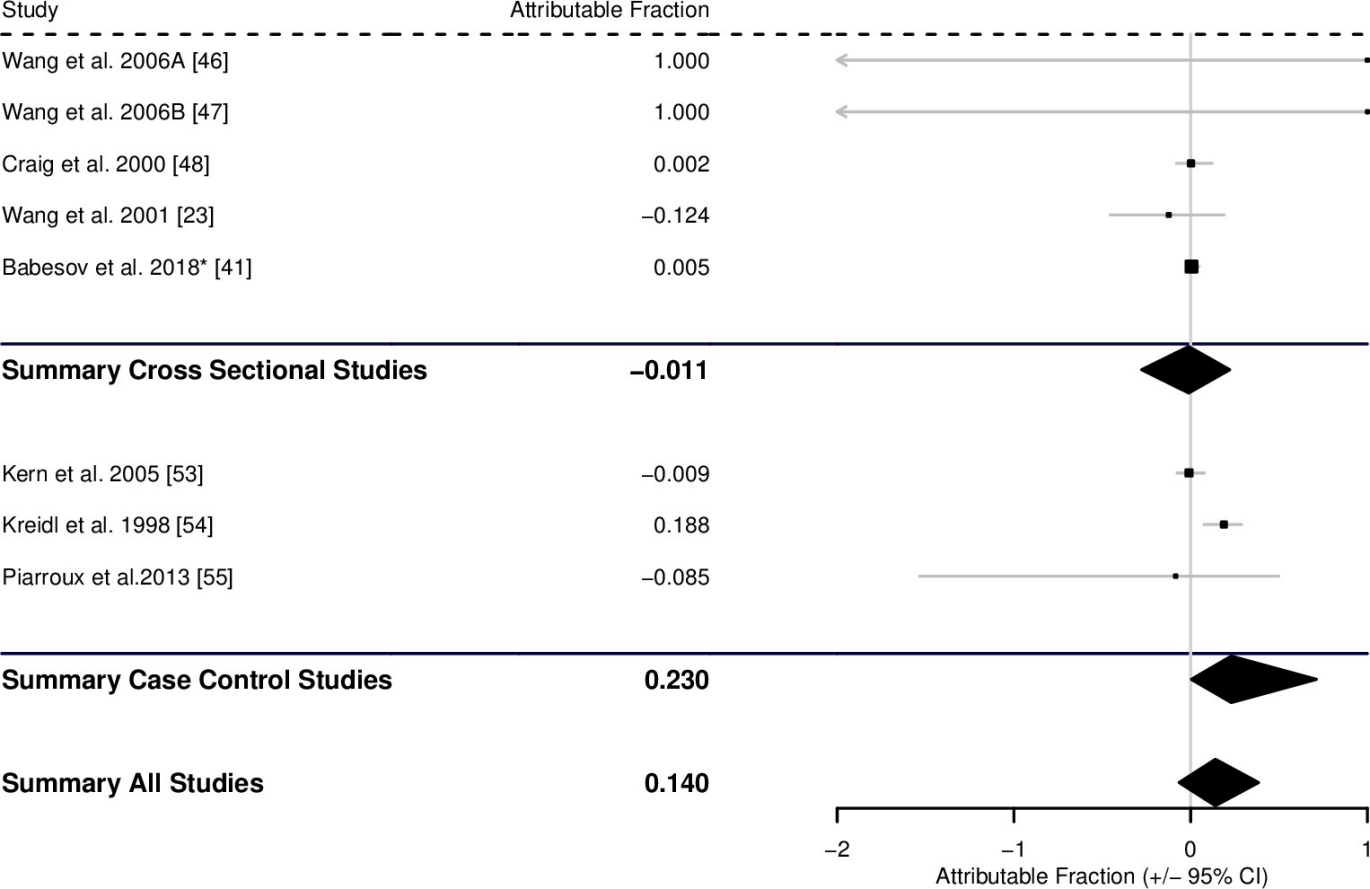
Forest plot for the attributable fraction for 5 cross-sectional studies and 3 case-control studies that analysed contact with foxes as a potential risk factor for human AE. *Data not reported in study, but supplied by the authors.

Regarding contact with infected foxes as a potential risk factor for infection with AE, there were 5 suitable cross-sectional studies giving an AF of -0.011 (-0.277–0.221), and 3 case-control studies giving an AF of 0.230 (0.004–0.712). This gave a pooled estimate of 0.140 (-0.065–0.384) (Fig 10, Table 1).

**Fig 11:**
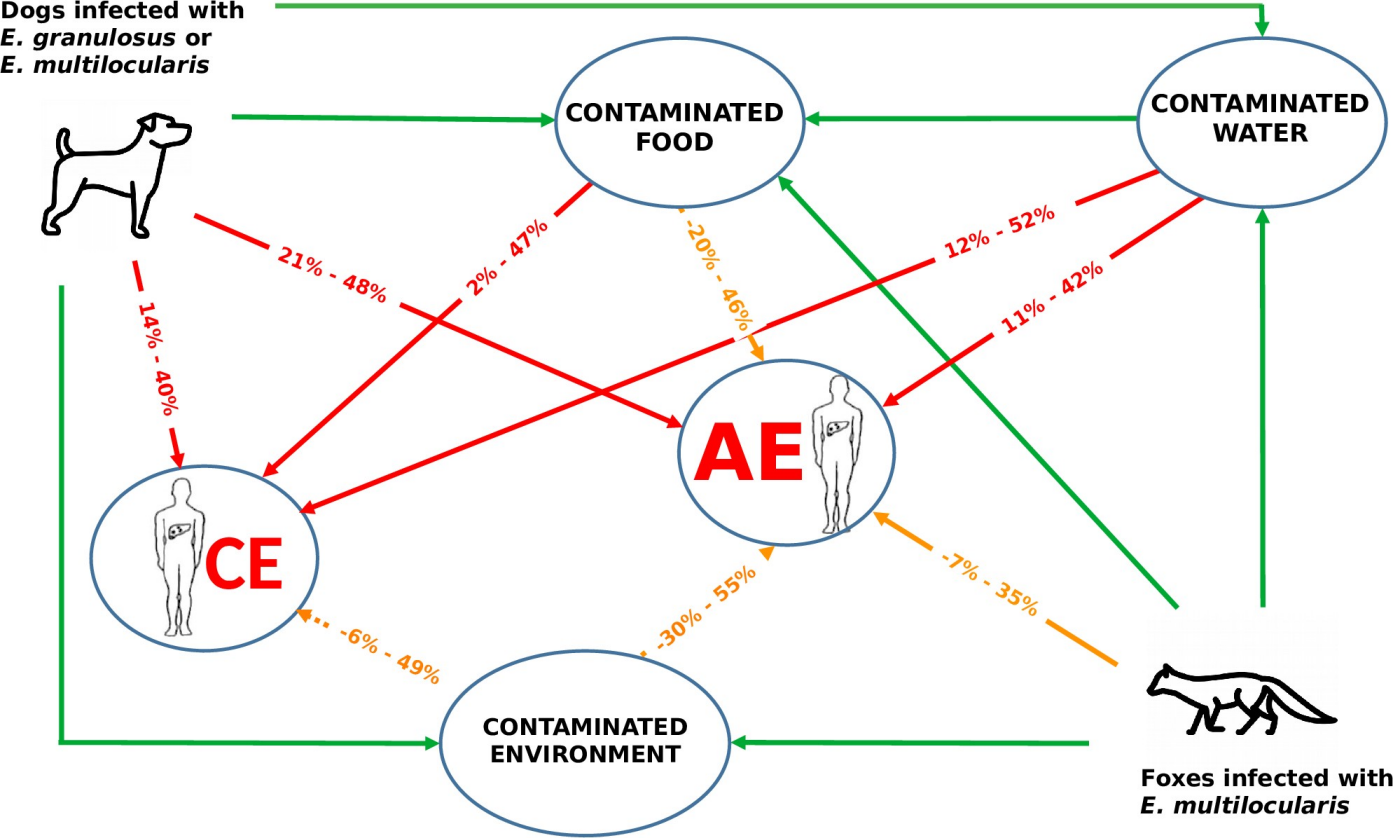
Transmission pathways for *E*. *granulosus* and *E*. *multilocularis* between the definitive host and humans. The 95% range (CI) for the attributable fraction of each pathway is given. Red lines indicate statistical evidence for the reported AF. Orange lines indicate insufficient statistical evidence (ie CI include 0). Dotted lines indicate the assumed pathway that is the residual AF after others are deducted. Green solid line indicates indirect pathway to human infection.

A diagrammatic summary of the transmission pathways considered and the AF for each is illustrated in Fig 11.

## Discussion

This study has attempted to estimate the attributable risk of a number of possible determinants (risk factors) of CE and AE. It should be emphasised that due to the small number of suitable reports from which data can be extracted, this is a global estimate. The determinant with the strongest evidence was contact with dogs and this was true for both CE and AE, at approximately 26% and 34%, respectively. That a high proportion of cases of CE was associated with dog contact was not a surprise, as dogs are the obligatory definitive hosts of *E*. *granulosus*; domestic dogs and livestock maintain the transmission cycle, with spill over to humans accounting for many cases of CE. In contrast, *E*. *multilocularis* has an important wildlife cycle between wild canids, especially foxes (*Vulpes* spp.), and small mammals (particularly arvicolids and other rodents), and this cycle is ecologically important in maintaining the endemicity of this parasite. However, high prevalences of *E*. *multilocularis* infection in dogs have been recorded in China and Kyrgyzstan (e.g., [57,58]) and these two countries also have the highest prevalences of human AE reported thus far.

There was evidence that contaminated water is a potential disease determinant for both CE and AE, with AF of approximately 29% and 25%, respectively. This epidemiological evidence is supported by the recent findings of *E*. *multilocularis* DNA in water sources [59], whilst taeniid eggs have been recovered from drinking water in Iran [60]. There is also the complication of ‘food crops eaten raw’ being watered, and/or washed, with contaminated water. The ultimate source of the parasite is, of course, the infected definitive host and contact with the definitive host should be seen as direct transmission. Other forms of transmission could be seen as indirect. There is a problem of distinguishing between ingestion of contaminated water and irrigation of crops with contaminated water, or food being washed in contaminated water. This shows that these two potential sources of disease may have some overlap that may not be precisely delineated in an epidemiology study. In the global burden of foodborne diseases study, foodborne illnesses were attributed to foods when they were caused by eating foods that were contaminated at the point at which they entered the place where they were prepared for final consumption [7]. Using this definition, disease from food that is contaminated by water through crop irrigation would be foodborne. But food contaminated by water at the place of consumption could be attributed to contaminated water.

For contaminated food, the evidence of this being a risk was less consistent. The pooled AF for CE was estimated at 23% and was significant (i.e. the CI did not include 0), but neither pooled AF for case-control nor cross-sectional studies considered separately were significant. Furthermore, the statistical approach utilized the trim and fill method to add in estimates for missing data and hence minimize bias. Without these estimates for missing data, the AF for contaminated food being a risk factor for CE would not be statistically different from zero (Table 1). For AE, the pooled AF across all studies was not statistically different from zero. However, considering the case-control studies alone, some evidence that food may be a pathway for infection did appear. It should be noted that the cross-sectional studies were all from China and central Asia where there are large numbers of cases of human AE. In contrast, 3 of the case-control studies were from Europe where the disease is rare. Hence, it could be argued that food may be an important transmission vehicle for this disease in Europe, but not elsewhere. This may reflect that relevant transmission routes elsewhere (such as, for example, waterborne transmission or, indeed, contact with infected dogs) may be less likely to occur in Europe where drinking water tends to be from a treated municipal supply, and treatment regimens for exposed dogs are often stringently applied. It is also important to note that the trim and fill method to estimate missing data indicated there was a missing study, which is why the pooled-adjusted AF is close to the highest of the individual studies. Taeniid eggs [61] have been isolated from raw salad vegetables so this transmission route can not be ruled out.

The issue of stray dogs can not be ignored. In many of the highly endemic regions for these two diseases, stray or feral dogs usually have a high prevalence of infection. This may lead to difficulties in interpreting the extent of dog contact as a transmission pathway. However, stray or feral dogs have, by definition, low rates of direct contact with humans, but may be important in contaminating food such as vegetables, water or the environment. Therefore, we would argue that the transmission to human from stray dogs is indirect and through these alternative pathways.

In the case of CE, suitable studies were found in Asia, especially China, and also Africa and Latin America, and thus the source attribution should be seen as having a global perspective. For AE, all the cross-sectional reports were from China, except one from Kyrgyzstan. In both these countries, endemic areas are characterized by a high prevalence of infection in dogs and a high prevalence of human AE. Thus, undertaking a cross-sectional study where human prevalences may be 5% or more is feasible and AF can hence be estimated from the potential risk factors examined, with the limitations that this applies to restricted geographical area. However, AE is generally a rare disease outside of Asia, and consequently epidemiological studies have used a case-control design. With the assumptions that OR is a reasonable estimate of RR and that the proportion of controls exposed to any risk factor is an estimate of such a proportion in the population, it has been possible to estimate the AF from such data. The cross-sectional studies and the case-control studies suggested an AF for exposure to infected dogs of 36% and 33% respectively, which shows substantial agreement between the different study types and endemic regions, although the epidemiology of transmission to humans may be different in Europe compared to that of Asia.

By performing this systematic review, we could only find relatively few studies with usable data. Given the long latent period between infection and the appearance of clinical signs, it is a challenge to identify possible determinants of disease. However, both contact with dogs and contaminated water were consistent findings, whilst that for contaminated food was inconsistent. Limits on the potential contribution by other determinants can be hypothesized, assuming these disease determinants are mutually exclusive (i.e., transmission from dogs and foxes, water, food and contaminated environment as a soil-borne helminth results in 100% of transmission). Thus, for CE, the combined AF due to dogs and water is approximately 56%, and similarly for AE it is approximately 59%. The expert knowledge elicitation of 21% for CE, as suggested by the global burden of foodborne diseases, is consistent with the empirical data presented here. However, the estimate of 47% for AE appears to be on the high side, as foodborne AE and contaminated environment would, between them, account for at most 41%, and the empirical evidence presented here did not find conclusive evidence to suggest that AE is substantially transmitted through food. There were very few data that specifically examined the risk of transmission through a contaminated environment. However assuming that the pathways are mutually exclusive then this would be represented by the residual attributable fraction: ie 21% for CE and 11% for AE (see Table 1).

These estimates all assume that the different pathways of transmission are mutually exclusive and hence the AF from these pathways would sum to 100%. It is also possible that the sum of AF due to various risk factors can be more than 100% [62]. This is because some individuals present with more than one risk factor. For example, gender is often associated with a diagnosis of echinococcosis possibly due to behavioural reasons. These can be hypothesised as one gender being more likely to care for the household dog. But it is the dog in which we are interested and the AF associated with dog contact would fall disproportionately on the gender in question. Thus, by exclusively investigating the four routes of transmission, rather than gender or occupational risk factors, the chance that the AF would sum to more than 100% should be reduced.

It should be noted that the AF contributed by food may be very different in different parts of the world. For example, AE is a rare disease in Europe and likely to have a different epidemiology of transmission than in China and Kyrgyzstan where the disease is more common. Due to this distribution of disease, case-control studies were more frequent from Europe and North America (endemic areas where the disease is rare), and cross-sectional studies predominated from China and Kyrgyzstan (endemic regions where the disease is more common). Thus, the AF estimated from the two different study designs may reflect the AF in low and high human incidence areas respectively. Only with more carefully designed studies can we achieve more accurate estimates of the source attribution of AE

Two systematic reviews on risk factors for CE and AE have been published recently [63,64], and we were able to identify a few additional studies that were published in Chinese, and a single study published in Russian, that were sourced through a multilingual search; thus our database appears to be relatively complete.

The results of this study confirm the importance of regular treatment of dogs with anthelmintics and other dog control measures as vitally important for control of CE. This is also true of AE in regions where there is a high human incidence of disease. Neither of these findings are surprising because of the nature of the life cycles of these two parasites. There are numerous reports on the control of echinococcosis and transmission models to predict the likely outcome of control measures (reviewed by Craig et al. 2017 [65]). These nearly all include regular anthelmintic treatment of dogs as a control measure. Our results indicate that access to safe drinking water and safe preparation of food could also ameliorate the risk of transmission to humans. However, this is only in the context where infected canids are contaminating water or food: i.e. in endemic regions. If there were no infected definitive hosts then water or food contamination would not occur.

The major weaknesses in this study is due to the relatively few data sets, especially regarding food as a source of infection for *Echinococcus*, which was the major research question of this study. Thus, the contribution of contaminated food remains uncertain, although it can be argued that the data presented here provide a better estimate than previous ones obtained via expert knowledge elicitation, as it is based on empirical evidence. Also, whilst most of the English language publications were peer-reviewed, this was less certain for some of the papers published in other languages, especially those in standard Chinese. It is therefore possible that the quality of the studies used in the meta-analysis was not consistent.

## Conclusion

Most underlying papers in the present meta-analysis originate from regions with high human incidence. Therefore the overall findings from such areas that, for both AE and CE, the largest AF is due to dog contact is quite strong and consistent. The evidence for water-transmitted echinococcosis is also convincing, albeit with a lower AF than dog contact. There is some evidence that food may be a source of echinococcosis, particularly in regions where disease burden is low, but the AF for CE is only significant when combining all studies: i.e. both cross sectional and case control studies. For AE in the regions of high human incidence there is little evidence for foodborne transmission. However in regions of low human incidence the evidence is more convincing.

## Supporting information

S1 Checklist PRISMA checklist. (DOC)
